# Influence of sintering temperature and Eu^3+^ concentration on the structural, optical, and Judd–Ofelt parameters of CaY_2_O_4_ phosphors synthesized by solid-state reaction

**DOI:** 10.1039/d6ra02573k

**Published:** 2026-07-02

**Authors:** Mai An Pham, Thi Kim Lien Vu, Ha Vi Can, Thanh Binh Nguyen, Kim Dan Ho, Thi Lien Pham, Anh Tuan Chu, Viet Ha Chu, Tien Ha Le

**Affiliations:** a Thai Nguyen University of Education Thai Nguyen 250000 Vietnam; b Institute of Theoretical and Applied Research, Duy Tan University Hanoi 100000 Vietnam; c Faculty of Natural Sciences, Duy Tan University Da Nang 550000 Vietnam; d Optical Materials Research Group, Science and Technology Advanced Institute, Van Lang University Ho Chi Minh City Vietnam; e Faculty of Applied Technology, Van Lang School of Technology, Van Lang University Ho Chi Minh City Vietnam; f Institute of Materials Science, Vietnam Academy of Science and Technology 18 Hoang Quoc Viet Cau Giay Hanoi Vietnam; g Viet Nam University of Traditional Medicine Hanoi 100000 Vietnam; h TNU – University of Sciences Thai Nguyen 250000 Vietnam halt@tnus.edu.vn letienha@tnu.edu.vn

## Abstract

In this study, we synthesized Eu^3+^-doped CaY_2_O_4_ materials using a solid-phase reaction method, with sintering temperatures ranging from 1000 to 1300 °C and Eu^3+^ concentrations ranging from 0 to 6%. SEM, XRD, Raman, FTIR, EDX, XPS, PL, and PLE measurements were used to examine the surface morphology, structure, microstructure, elemental ratios, surface ionic bonding, and optical properties of the materials. The results show that the fabricated materials have an orthorhombic structure in the *Cmcm* space group (No. 63), with the highest crystallinity achieved at 1200 °C. As the sintering temperature increased from 1000 to 1200 °C, particle size increased from 100 nm to 800 nm, and aggregation into larger particles began at 1300 °C. At the same time, the crystal size decreased from 49.53 nm to 37.00 nm, corresponding to an increase in the Eu^3+^ concentration from 2% to 6%. Optical property analysis indicated that the material exhibits strong absorption in the ultraviolet and near-ultraviolet regions, resulting in intense red emission with characteristic Eu^3+^ ion lines during transitions from the excited state ^5^D_0_ to the ^7^F_*j*_ states. Concentration-induced fluorescence quenching was also detected at 5% Eu^3+^ concentration. The analysis suggests that this material is suitable for applications as a color display material and as a red-emitting component in white LEDs.

## Abstract 2

In this study, we synthesized Eu^3+^-doped CaY_2_O_4_ materials using a solid-phase reaction method, with sintering temperatures ranging from 1000 to 1300 °C and Eu^3+^ concentrations ranging from 0 to 6%.

## Graphical abstract

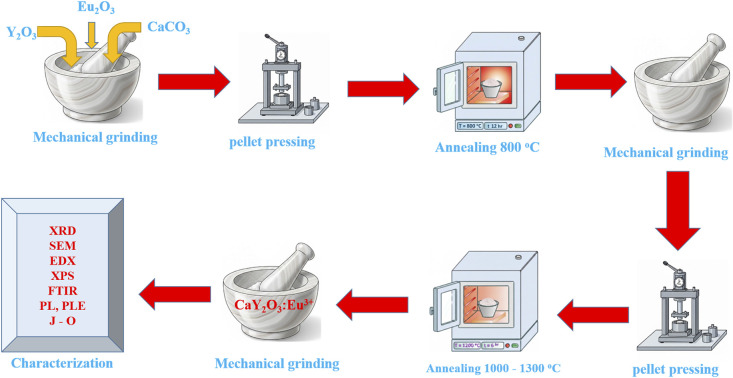

## 1. Introduction

In recent decades, the rapid development of solid-state lighting technology and next-generation color display devices has spurred research into luminescent materials with high quantum efficiency and good thermal stability.^1,2^ Among red luminescent materials, trivalent rare earth ions and transition metals have attracted considerable attention from researchers. Among rare earth ions capable of emitting red light, the Eu^3+^ ion is particularly noteworthy due to its strong emission in the red light region with high color purity and its good absorption of radiation in the near ultraviolet and blue light regions, making it highly suitable for applications in LED fabrication using UV-LED or Blue-LED excitation chips.^1,3–8^

To ensure that the Eu^3+^ ion effectively performs its emissive role in the energy transition from the excited state to a lower-energy state with high efficiency and good thermal stability, finding suitable substrates for Eu^3+^ doping is essential. Studies have shown that the AB_2_O_4_ spinel-structured material system exhibits superior properties, including high thermal stability and an octahedral [BO_6_] cluster, which are highly conducive to doping rare-earth ions to enhance their optical properties.^6–10^ Therefore, this spinel family is widely used in many studies as a substrate for rare-earth-ion doping in various fields, such as radiation dosimetry, photothermal materials, and color display devices.^14–17^

Studies have shown that the CaY_2_O_4_ substrate system, with an orthorhombic structure in the *Cmcn* space group (No. 63), a stable crystal lattice, and low phonon energy, minimizes non-radiative recovery processes, thereby improving the luminescence efficiency of rare-earth ions when doped into this substrate. In particular, in the CaY_2_O_4_ substrate, Y^3+^ ions occupy two non-equivalent crystal positions with low central symmetry (Cs)^1^. When these positions are replaced with Eu^3+^ ions, the lack of inversion centers at these lattice sites enhances the optical properties of the material. As a result, CaY_2_O_4_ : Eu^3+^ materials exhibit a very high asymmetry ratio (*R*/*O* ratio), producing red light with superior color purity, often exceeding 95%, meeting the stringent standards for color display devices and near-UV-stimulated white LEDs (WLEDs).^18^

Although CaY_2_O_4_ systems exhibit many superior properties for use as color display materials when doped with rare-earth ions, the CaY_2_O_4_ system doped with Eu^3+^ still presents several issues that require further study, such as the influence of the doping ion concentration on the material's optical and crystallographic parameters.^11–13^

This study investigates the influence of sintering temperature and Eu^3+^ ion concentration on the material's structure, optical properties, and optical parameters. Discoveries into the influence of Eu^3+^ ions on the crystal growth of the material, as well as their impact on optical parameters, will help us better understand the optical mechanisms in advanced materials, thereby potentially opening avenues for doping and co-doping different ions on the same substrate material.

## 2. Materials and methods

### 2.1. Materials

**Fig. 1:**
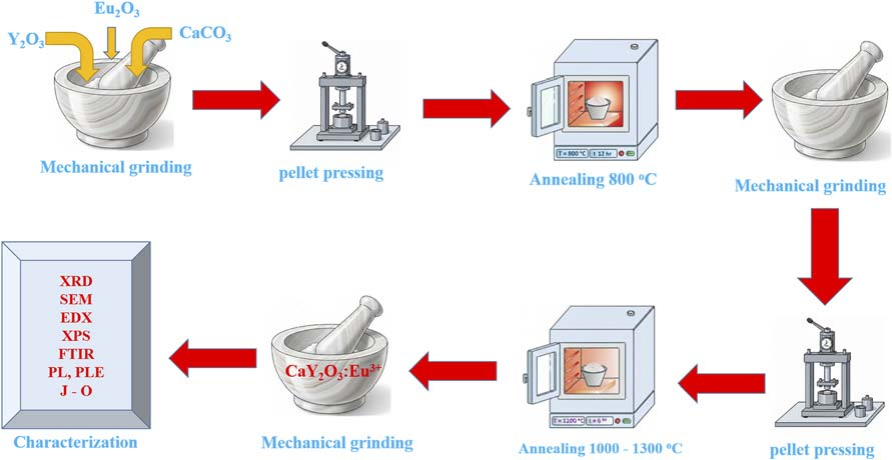
Schematic illustration of the solid-state synthesis process of CaY_2_O_4_ : Eu^3+^ phosphors and subsequent characterization of their material properties.

A series of CaY_2_O_4_ : *x*% Eu^3+^ (*x* = 0.0–6) samples was generated using a classic solid-state reaction method. Firstly, stoichiometric amounts of CaCO_3_ (purity >99.9%), yttrium oxide (Y_2_O_3_, purity >99.9%), and europium(iii) (Eu_2_O_3_, purity >99.9%) were obtained. Initially, the material was hand-ground using an agate mortar in C_2_H_5_OH, then dried at 90 °C for 5 hours, followed by pelleting and pre-firing at 800 °C for 12 hours. After pre-firing, the material was ground a second time and sintered at 1000–1300 °C in air for 9 hours, with a heating rate of 300 °C hour^−1^, and then naturally cooled to obtain fluorescent CaY_2_O_4_ powder doped with Eu. The material was then ground into powder for measurement and analysis of its properties. The material synthesis procedure and material property analysis are shown in Fig. 1.

### 2.2. Characterization

The crystal structure of the samples was analyzed using an X-ray diffraction (XRD) pattern over 20–80° 2*θ* on an XRD D2 Advanced instrument. This analysis was simultaneously supported by a transmission electron microscope (JEM-2500SE, JEOL) operating at 200 kV. Raman spectra were used to study the vibrational modes and phase composition of the materials (XploRa Plus, France, 2019) with a 785 nm laser excitation source. The samples' surface morphology and elemental composition were observed using field-emission scanning electron microscopy (FESEM) and energy-dispersive spectroscopy (EDS) on a JSM-7600F instrument. The bonding energy of ions in the investigated sample was analyzed using XPS spectra obtained with a XPS spectrometer (XPS, Thermo Scientific, USA) equipped with a Al Kα source. The optical properties of the samples were investigated using PL and PLE spectra obtained with a PL spectrophotometer equipped with a 450 W Xenon discharge lamp as the excitation source.

## 3. Results and discussion

### 3.1. Morphology

**Fig. 2:**
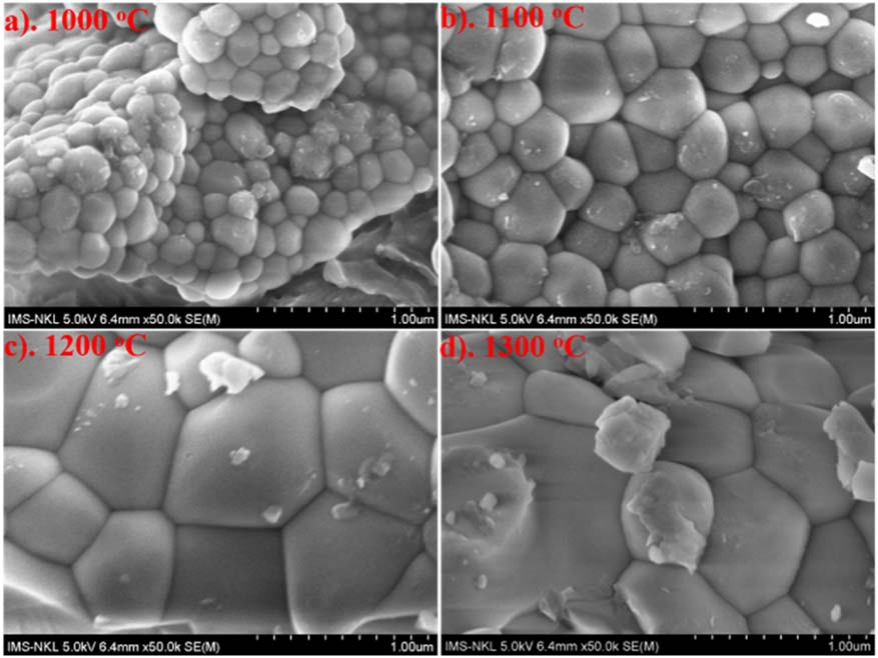
SEM measurement results of CaY_2_O_4_ : 5% Eu^3+^ with sintering temperature from 1000–1300 °C.

Fig. 2 shows SEM images of CaY_2_O_4_ : 5% Eu^3+^ samples with sintering temperatures ranging from 1000 to 1300 °C. Analysis results show that as the sintering temperature increases, the particle size of the resulting material increases: at 1000 °C, the average particle size is approximately 100 nm; at 1100 °C, the average size is approximately 300 nm; at 1200 °C, the average size is approximately 800 nm; and there is a tendency for agglomeration when the sintering temperature reaches 1300 °C. This indicates that as the sintering temperature increases, small particles combine to form larger particles and simultaneously tend to clump together into bulk-like materials. This analysis shows that to obtain a material with large particles and a smooth surface, it must be sintered at 1200 °C.

### 3.2. XRD patterns

**Fig. 3:**
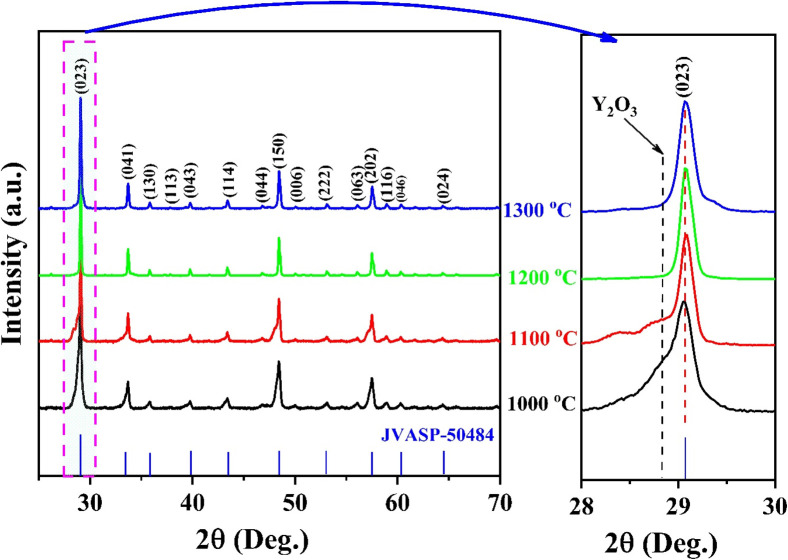
X-ray diffraction pattern of a CaY_2_O_4_ sample doped with 5% Eu and sintered at temperatures of 1000–1300 °C.

To study the effects of sintering temperature and Eu^3+^ ion doping concentration on the material's crystal structure, we performed X-ray diffraction on the prepared samples. Fig. 3 shows the X-ray diffraction spectra of CYO samples doped with 5% Eu^3+^ ions at sintering temperatures from 1000 to 1300 °C. The results show that at 1000 °C the material formed an orthorhombic CaY_2_O_4_ structure belonging to the *Cmcm*, 63 space group corresponding to the theoretically calculated structure JARVIS-ID:JVASP-50484 with characteristic diffraction peaks at positions 29.308, 33.942, 35.807, 39.787, 43.435, 48.495 and 57.668° corresponding to the crystal lattice planes (023), (041), (130), (043), (150) and (202). Results on this structural phase of CaY_2_O_4_ have been reported in some recent publications,^1,18^ but this issue remains unclear because these X-ray diffraction peaks are close to the characteristic peaks of cubic Y_2_O_3_. According to the XRD analysis at 1000 and 1100 °C, the sample still contained the initial precursors CaO and Y_2_O_3_. Therefore, a broad shoulder was observed in the 2*θ* region (28 to 30°), representing the overlapping of two characteristic diffraction peaks of Y_2_O_3_ and orthorhombic CaY_2_O_4_ in this region. As the sintering temperature increased, the reaction between the precursors CaO and Y_2_O_3_ in the material occurred with higher efficiency, and the sample was almost entirely composed of the CaY_2_O_4_ structural phase. When the sintering temperature reached 1200 °C, the Y_2_O_3_ structural phase peak in the 2*θ* diffraction angle region (28 to 30°) was no longer observed. This indicates that at this temperature the precursors have combined almost completely, and the resulting material is relatively pure, with the main diffraction peaks corresponding to an orthorhombic CaY_2_O_4_ structure in the *Cmcm* space group (No. 63). At 1300 °C, the diffraction peak shifts to a higher 2*θ* angle and the half-width increases, while a shoulder appears at 29.38°. The above analysis shows that the resulting material has an orthorhombic structure belonging to the *Cmcm* space group (No. 63) and yields the best crystal quality when sintered at 1200 °C.

**Fig. 4:**
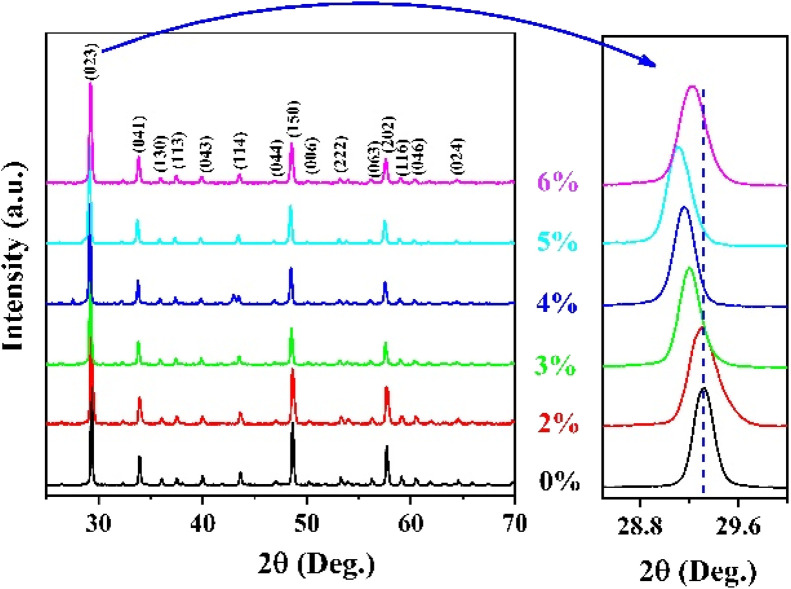
X-ray diffraction pattern of CaY_2_O_4_ : *x*% Eu^3+^ sample calcined at 1200 °C with different concentrations.

Based on the optimization of sintering conditions, CaY_2_O_4_ : *x*Eu^3+^ samples with Eu^3+^ concentrations from 0 to 6% were synthesized at 1200 °C in air for 9 hours to study how activator concentration affects structural and optical properties. Fig. 4 shows the X-ray diffraction patterns of these samples. All diffraction peaks correspond to the orthorhombic CaY_2_O_4_ phase with the *Cmcm* space group (No. 63), and no impurity phases are observed within the resolution of the XRD measurement. This suggests that Eu^3+^ ions were successfully incorporated into the CaY_2_O_4_ host lattice, likely by replacing Y^3+^ sites, without significantly changing the main crystal structure.

Although the overall diffraction pattern remains largely unchanged as the Eu^3+^ concentration increases, a slight shift of several diffraction peaks toward lower 2*θ* values is observed at low doping levels. This shift indicates an increase in the interplanar spacing of the lattice. Since the ionic radius of Eu^3+^ is larger than that of Y^3+^, partially replacing Y^3+^ with Eu^3+^ can cause local lattice expansion. According to Bragg's law, a larger *d*-spacing results in a decrease in the diffraction angle. Therefore, the low-angle shift further confirms the successful incorporation of Eu^3+^ ions into the CaY_2_O_4_ lattice at low doping levels. However, at higher Eu^3+^ concentrations, the peak positions stay relatively stable but shifted more toward lower 2*θ* values, suggesting that the host lattice can only accommodate a limited amount of Eu^3+^ before lattice distortion or defect effects become more prominent.

To more accurately assess this observation, we calculated the crystal size of the material using Scherrer's formula:1
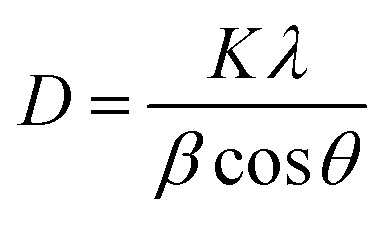
where *D* is the average crystallite size, *K* is the dimensionless constant value (0.89), *λ* = 1.540 Å is the wavelength of the irradiation, and *β* is the full width at half maximum measured in radians. Using eqn (1), we determined the crystal sizes of CaY_2_O_4_ samples doped with *x*% Eu^3+^ ions (*x* = 0, 2, 3, 4, 5, 6) to be 49.53, 45.94, 44.97, 44.56, 44.30, and 37.00 nm, respectively. These calculation results are shown in Table 1.

**Table 1:** XRD parameters, including 2*θ* position, FWHM, and average crystallite size of CaY_2_O_4_ : *x*% Eu^3+^ phosphors calcined at 1200 °C, estimated using the Scherrer equation

Samples	2*θ* (deg)	FWHM (deg)	Crystallite size in (023) direction (nm)
CaY_2_O_4_ : 0% Eu^3+^	29.3188	0.1639	**49.53**
CaY_2_O_4_ : 2% Eu^3+^	29.2927	0.1767	**45.94**
CaY_2_O_4_ : 3% Eu^3+^	29.2063	0.1805	**44.97**
CaY_2_O_4_ : 4% Eu^3+^	29.1607	0.1821	**44.56**
CaY_2_O_4_ : 5% Eu^3+^	29.1194	0.1874	**43.30**
CaY_2_O_4_ : 6% Eu^3+^	29.2297	0.2194	**37.00**

As summarized in Table 1, the average crystallite size calculated from the Scherrer equation exhibits a downward trend with increasing Eu^3+^ concentration. This decrease in crystallite size does not correlate with the ionic radius expansion; instead, it reflects the altered crystal growth kinetics induced by dopant incorporation. The substitution of Y^3+^ ions by the larger Eu^3+^ ions introduces local lattice distortion and microstrain within the CaY_2_O_4_ host matrix. These structural perturbations act as defects that suppress grain boundary mobility and impede atomic diffusion during the calcination process. Furthermore, a higher dopant concentration increases the density of local nucleation centers, leading to defect-limited growth and the subsequent formation of smaller coherent scattering domains. This refinement of crystallite size with heavily doped trivalent rare-earth ions aligns well with behaviors documented in established literature.

**Fig. 5:**
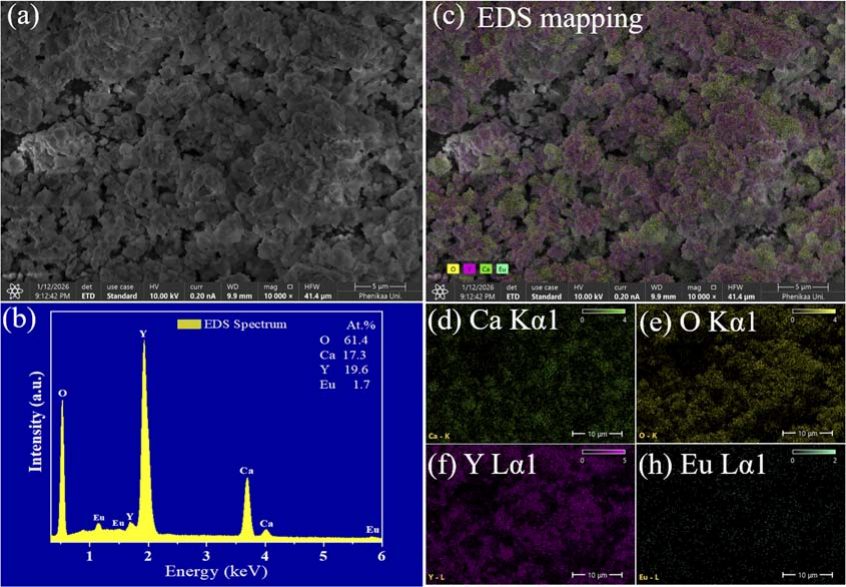
SEM and EDX images of the CaY_2_O_4_ : 5% Eu^3+^ sample calcined at 1200 °C.

To further examine the elemental composition and spatial distribution of the synthesized phosphor, EDS analysis was performed on the CaY_2_O_4_ : 5% Eu^3+^ sample. As shown in Fig. 5b, the EDS spectrum confirms the presence of Ca, Y, O, and Eu elements in the analyzed region, with measured atomic percentages of 17.3%, 19.6%, 61.4%, and 1.7%, respectively. No obvious foreign elements were detected within the detection limit of the EDS measurement, indicating that the sample mainly consists of the expected CaY_2_O_4_ : Eu^3+^ components. The corresponding elemental mapping images in Fig. 5c–h further show that Ca, Y, O, and Eu are distributed throughout the selected area, suggesting a relatively uniform elemental distribution at the observed scale.

For comparison, if Eu^3+^ is assumed to substitute for Y^3+^ sites in the CaY_2_O_4_ host lattice, the nominal composition of the 5% Eu^3+^ doped sample can be approximately expressed as CaY_1.9_Eu_0.1_O_4_. Based on this nominal formula, the theoretical atomic percentages of Ca, Y, Eu, and O are approximately 14.3%, 27.1%, 1.4%, and 57.1%, respectively. Compared with these theoretical values, the EDS result shows slightly higher Ca and Eu contents, while the Y content is lower than the nominal value. This deviation does not necessarily indicate the formation of impurity phases, because EDS provides only local and semi-quantitative compositional information from a limited surface region of the sample.

The higher apparent Ca and Eu contents may be related to several factors. First, the EDS signal is collected from a selected microscopic region and may not fully represent the average bulk composition of the whole sample. Local compositional fluctuations can occur in solid-state synthesized materials due to incomplete diffusion or local enrichment of certain elements. Second, Eu^3+^ ions may not be perfectly and uniformly incorporated into the CaY_2_O_4_ lattice. A small fraction of Eu-containing species may be enriched near particle surfaces, grain boundaries, or defect-rich regions, leading to a higher measured Eu content in the selected EDS area. Third, the surface morphology, roughness, particle agglomeration, and interaction volume of the electron beam can influence the detected X-ray intensity and the calculated atomic percentages. In addition, quantitative analysis of light elements, such as oxygen, by EDS is generally less reliable, and any uncertainty in oxygen quantification can affect the normalized atomic percentages of heavier elements, such as Ca, Y, and Eu.

Therefore, the EDS results should be interpreted as qualitative and semi-quantitative evidence for the presence and spatial distribution of Ca, Y, O, and Eu in the synthesized CaY_2_O_4_ : Eu^3+^ phosphor, rather than as an exact determination of the stoichiometric composition. Together with the XRD results, which show the formation of the orthorhombic CaY_2_O_4_ phase without detectable secondary phases, the EDS and elemental mapping results support the successful introduction of Eu^3+^ into the CaY_2_O_4_-based material system.

### 3.3. Effect of Eu^3+^ ion concentration on infrared absorption

**Fig. 6:**
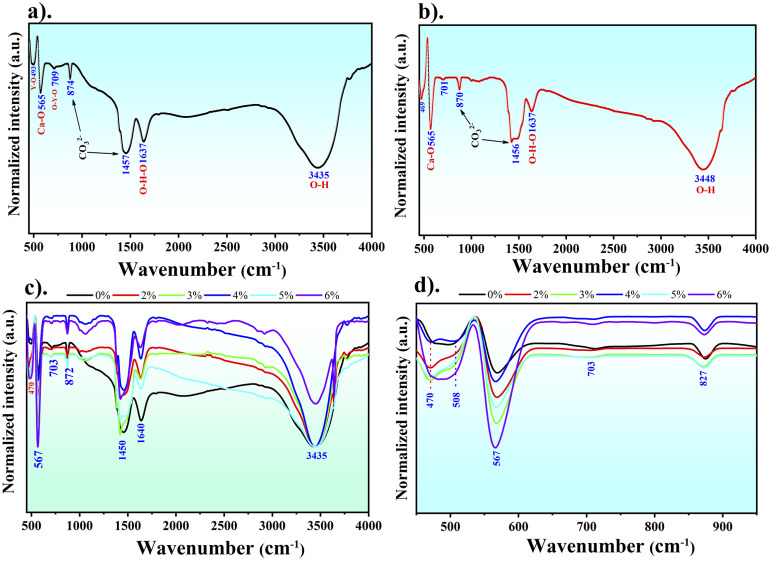
FTIR spectra of CaY_2_O_4_ : 0% Eu^3+^ samples (a), CaY_2_O_4_ : 5% Eu^3+^ samples (b), and CaY_2_O_4_ : *x*% Eu^3+^ samples (*x* = 0–6%) taken from 450–4000 cm^−1^ (c), and scanned from 450–1000 cm^−1^ (d).

To further evaluate the effect of Eu^3+^ doping ion concentration on the chemical bonding environment of the host CaY_2_O_4_ lattice, FTIR spectroscopy was performed on CaY_2_O_4_ : *x*Eu^3+^ samples calcined at 1200 °C, with Eu^3+^ concentrations ranging from 0 to 6%. As shown in Fig. 6a, the undoped CaY_2_O_4_ sample showed several absorption bands located at approximately 493, 565, 709, 874, 1457, 1673, and 3435 cm^−1^. The absorption bands in the low wavelength region are mainly associated with lattice vibrations characteristic of the host CaY_2_O_4_ lattice, including vibrational modes related to Y–O, Ca–O, and O–Y–O. The absorption bands observed at higher wavelengths can be attributed to surface adsorbents, such as C–O vibrations from atmospheric CO_2_ and O–H/H–O–H vibrations from adsorbed water molecules.

As shown in Fig. 6b–d, no additional anomalous absorption bands were observed after Eu^3+^ ion doping, indicating that Eu^3+^ incorporation does not generate foreign functional groups or detectable secondary phases within the sensitive range of FT-IR analysis. Compared to the undoped CaY_2_O_4_ sample, the absorption bands associated with Y–O and O–Y–O vibrations show a slight shift towards lower wavelengths upon Eu^3+^ incorporation. This shift is thought to occur when Eu, with its larger atomic mass, replaces the position of the smaller Y atom, altering the reduced mass of the system around the replaced position. It causes the vibrational frequency of the metal–oxygen bond, governed by both the reduced mass of the vibrator and the corresponding bond strength constant, to change. Therefore, the observed shift is related to changes in the local coordination environment, bond length, bond strength, and lattice distortion due to the partial replacement of Y^3+^ with Eu^3+^ in the CaY_2_O_4_ crystal lattice.

Since Eu^3+^ has a larger ionic radius than Y^3+^, its incorporation into the positions of Y^3+^ can locally expand the surrounding oxygen coordination environment, slightly elongating the Eu/Y–O bonds and altering the bond stiffness. The combined effect of increasing the reduced mass and decreasing the local binding force constant can lead to a decrease in vibrational frequency, resulting in the observed shift of the Y–O and O–Y–O associated bands towards lower wavenumbers. Conversely, the absorption band associated with the Ca–O vibration remained virtually unchanged after Eu^3+^ doping, suggesting that the Ca-associated binding environment was only slightly affected.^1,18^

FTIR analysis results show that the incorporation of Eu^3+^ primarily affects the Y–O associated local structure rather than the Ca–O binding environment. This observation supports the hypothesis that Eu^3+^ ions preferentially replace Y^3+^ sites in the CaY_2_O_4_ crystal lattice. This result is consistent with our previous analyses regarding the substitution ability of Eu^3+^ ions in the CaY_2_O_4_ crystal lattice.

### 3.4. XPS spectra investigation

**Fig. 7:**
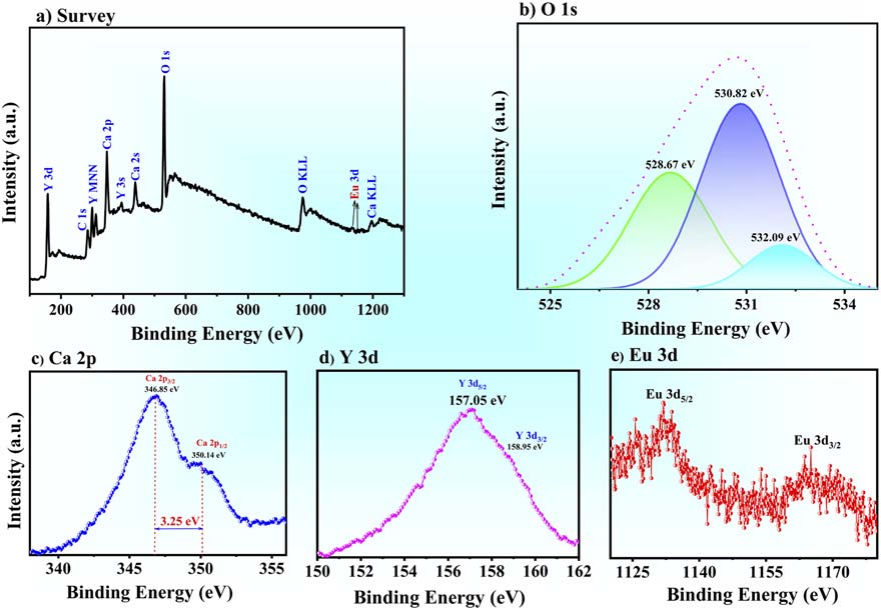
XPS spectra of CaY_2_O_4_ doped with 5% Eu (a), high-resolution XPS spectra of O 1s (b), Ca 2p (c), Y 3d (d), and Eu 3d (e).

To better determine the chemical bonding state of Eu^3+^-doped CaY_2_O_4_ material, we performed XPS measurements on sintered CaY_2_O_4_ samples doped with 5% Eu^3+^ ions at 1200 °C. Fig. 7 presents the XPS spectra of the sintered CaY_2_O_4_ samples doped with 5% Eu^3+^ ions at 1200 °C, along with high-resolution XPS spectra for the O 1s, Ca 2p, Y 3d, and Eu 3d states of the ions present in the CaY_2_O_4_ : 5% Eu sample, and the C 1s state signal from the measuring instrument. These measurement results were normalized by C 1s (284.7 eV). Fig. 7b shows the high-resolution XPS spectrum of the O 1s state, characteristic of the O^2−^ ion at 530.63 eV, with a spectral width of approximately 10 eV.

The high-resolution O 1s spectrum was deconvoluted into three distinct sub-peaks located at 528.67, 530.82, and 532.09 eV. The major peak at 528.67 eV corresponds to the lattice oxygen O^2−^ bound to the metal cations Ca^2+^, Y^3+^, and Eu^3+^ within the host matrix. The intermediate peak observed at 530.82 eV is ascribed to an oxygen-deficient or defect-related oxygen environment within the CaY_2_O_4_ crystal lattice. While this region is traditionally linked to the formation of oxygen vacancies, in the context of *ex situ* powder analysis, it more accurately reflects a highly disordered local coordination sphere resulting from structural defects. Finally, the highest binding energy peak at 532.09 eV is appropriately attributed to surface hydroxyl groups or chemisorbed water species on the material surface. The binding state of the Ca^2+^ ion is shown in Fig. 7c as the Ca 2p state with two peaks at 346.85 and 350.14 eV, corresponding to the Ca 2p_3/2_ and Ca 2p_1/2_ states characteristic of the Ca^2+^ ion. These two peaks have energy splitting up to 3.25 eV. Fig. 7d shows the binding state of the Y^3+^ ion in the corresponding Y 3d state, with a peak at 157.05 eV and a shoulder at 158.95 eV. These XPS peaks and shoulders are believed to be the Y 3d_5/2_ and Y 3d_3/2_ states characteristic of the Y^3+^ ion. Fig. 7e shows a high-resolution XPS spectrum of the Eu^3+^ ion in the sample, with two peaks at 1132.27 and 1166.94 eV, corresponding to the 3d_5/2_ and 3d_3/2_ states, respectively. The presence of these two XPS peaks demonstrates the successful incorporation of Eu into the CaY_2_O_4_ matrix.

### 3.5. Optical properties

**Fig. 8:**
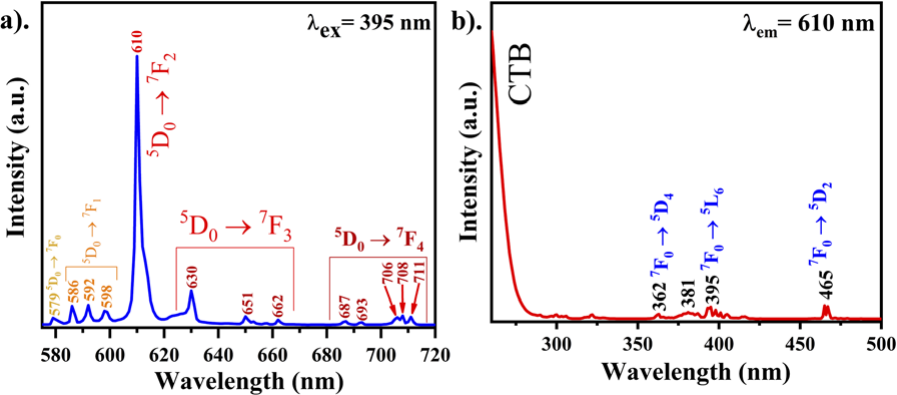
The fluorescence excitation spectra at 395 nm (a), and the fluorescence excitation corresponding to the emission peak at 610 nm (b) of the 5% Eu^3+^-doped CaY_2_O_4_ sample, calcined at 1200 °C and measured at room temperature.

The analysis results above show that the material crystallizes best at 1200 °C. Therefore, we conducted a fluorescence spectrum survey of the CaY_2_O_4_ : 5% Eu sample, sintered at 1200 °C, with an excitation wavelength of 395 nm. The results in Fig. 8a show that the material emits in the orange-to-red region, with characteristic Eu^3+^ emissions at 579, 586, 592, 598, 610, 630, 651, 662, 687, 693, 706, 708, and 711 nm. The emission peak at 579 nm is assigned to the ^5^D_0_ → ^7^F_0_ transition. The peaks at 586, 592, and 598 nm are assigned to the ^5^D_0_ → ^7^F_1_ transition. The most intense emission peak at 610 nm is attributed to the electric dipole ^5^D_0_ → ^7^F_2_ transition. The peaks at 630, 651, and 662 nm are assigned to the ^5^D_0_ → ^7^F_3_ transition, while the emission peaks at 687, 693, 706, 708, and 711 nm are assigned to the ^5^D_0_ → ^7^F_0_ transition of Eu^3+^ ions in the CaY_2_O_4_ host matrix. Among these emission peaks, the one at 610 nm has the highest intensity, corresponding to the electric dipole (ED) transition from ^5^D_0_ to ^7^F_2_. This indicates that Eu^3+^ ions occupy asymmetrical positions within the CaY_2_O_4_ crystal lattice.^19–21^

To investigate the absorption capacity of this material, we performed fluorescence excitation spectroscopy, with the strongest emission peak at 610 nm, and obtained the results shown in Fig. 8b. The analysis results show that the material strongly absorbs in the near-ultraviolet and blue regions. The strong ultraviolet absorption band below 280 nm is attributed to charge-transfer transitions in Eu^3+^ ions. The sharp excitation peaks at 362, 381, 395, and 465 nm correspond to the absorption of Eu^3+^ ions from the ground state ^7^F_0_ to higher-energy states ^5^D_4_, ^5^G_J_, ^5^L_6_, and ^5^D_2_.

**Fig. 9:**
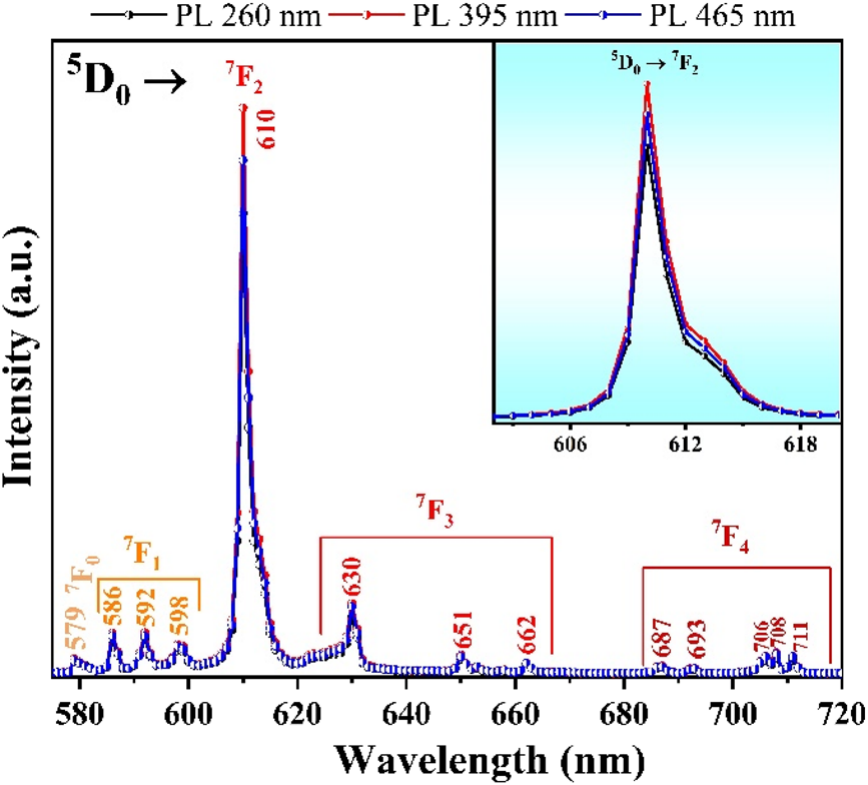
Fluorescence spectra of the CaY_2_O_4_ : 5% Eu sample at different excitation wavelengths.

To select the appropriate excitation wavelength for the material's luminescence and thereby guide its application across different fields, we measured its fluorescence spectrum for the aforementioned excitation bands and peaks. Fig. 9 shows the fluorescence spectrum of the CaY_2_O_4_ : 5% Eu material sample calcined at 1200 °C with different excitation wavelengths of 260, 395, and 465 nm. The observed results show that the material emits in the high-red region, with characteristic emission peaks of Eu^3+^ ions when transitioning from the ^5^D_0_ excited state to the ^7^F_*J*_ states (*J* = 0, 1, 2, 3, 4) in the CaY_2_O_4_ matrix. The emission peaks did not change position when excited at different wavelengths, and the excitation wavelength for the strongest emission was 395 nm, corresponding to the absorption of Eu^3+^ ions from the ground state ^7^F_0_ to the excited state ^5^L_6_. This result is consistent with findings from other research groups studying Eu^3+^-doped spinel materials for red-light emission.

**Fig. 10:**
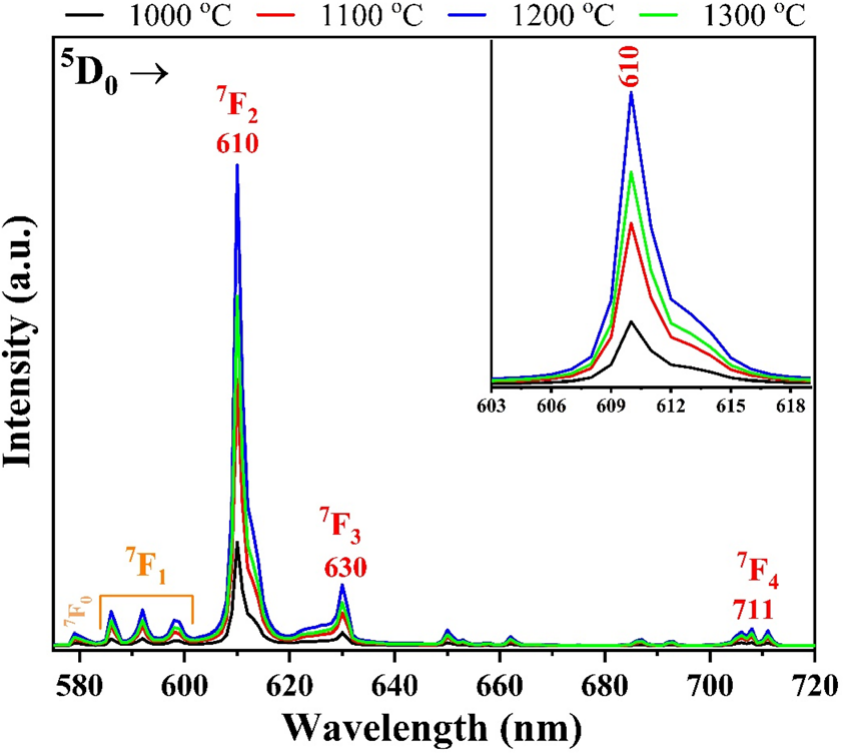
Fluorescence spectra of CaY_2_O_4_ : 5% Eu samples excited at 395 nm with different sintering temperatures.

Because the material exhibits the strongest emission in the red region upon excitation at 395 nm, we used this wavelength to investigate the effects of sintering temperature and Eu^3+^ ion concentration on the material's emission. Fig. 10 shows the fluorescence spectra of CaY_2_O_4_ : 5% Eu^3+^ samples with sintering temperatures ranging from 1000 to 1300 °C. The results show that the material emits strongly in the orange-red region, with characteristic emission peaks corresponding to energy-level transitions of Eu^3+^ ions from the ^5^D_0_ state to the ^7^F_*J*_ state. The positions of these emission peaks remain almost unchanged as the sintering temperature increases. As the sintering temperature increases, the fluorescence peak intensities tend to increase and reach a maximum at 1200 °C.

This result is explained by the fact that as the sintering temperature increases, the crystal quality of the CaY_2_O_4_ matrix improves, the particle size increases, reducing surface scattering of the material. Simultaneously, increasing the sintering temperature enhances Eu diffusion into the matrix, thereby increasing the concentration of emission centers and the material's fluorescence intensity. When the sintering temperature increases to 1300 °C, crystal quality decreases, leading to a reduction in the material's fluorescence intensity. This analysis is consistent with the investigation of the material's surface morphology and crystal structure in Sections 3.1 and 3.2.

**Fig. 11:**
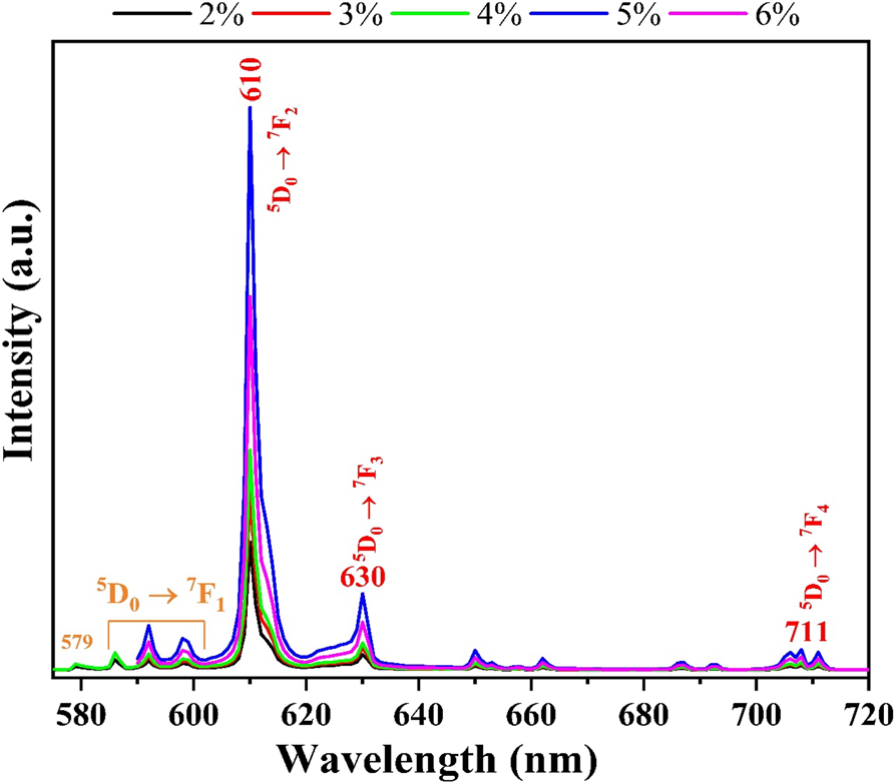
PL of CaY_2_O_4_ samples doped with Eu at different concentrations, with excitation wavelength of 395 nm.

Based on the fact that the material exhibits the best emission at a sintering temperature of 1200 °C and an excitation wavelength of 395 nm, we investigated the effect of Eu^3+^ ion concentration on the optical properties of this material. Fig. 11 shows the fluorescence spectra of CaY_2_O_4_:Eu samples with doping concentrations ranging from 2 to 6%, sintered at 1200 °C, and excited at 395 nm. The results show that as the Eu^3+^ ion concentration increases, the peak fluorescence intensity increases to a maximum at 5% doping concentration, then decreases. This result indicates that as the doping concentration increases, the density of emission centers generated by replacing Y^3+^ ions with Eu^3+^ ions increases, thereby increasing fluorescence intensity. However, when the ion concentration exceeds 5%, it leads to fluorescence quenching due to a concentration gradient induced by energy transfer between emission centers, which increases non-radiative recombination and decreases fluorescence intensity.^18,22^

Changes in Eu^3+^ ion concentration affect the local crystal field around Eu when replacing Y^3+^ ions. Studies show that in f–f transitions of the ^4^fn configuration of Eu^3+^ ions, the dipole transition from the ^5^D_0_ state to the ^7^F_2_ state is highly sensitive to the crystal field, while the dipole transition from the ^5^D_0_ state to the ^7^F_1_ state remains virtually unchanged when the surrounding environment of Eu^3+^ ions changes. To evaluate this change, we investigated the ratio between the intensity of the ^5^D_0_ – ^7^F_2_ and ^5^D_0_ – ^7^F_1_ (*R*) shifts to investigate the effect of Eu^3+^ ion doping concentration on the ionization of Eu–O. This *R* ratio was determined by formula (2), and the results are obtained in Table 3:2
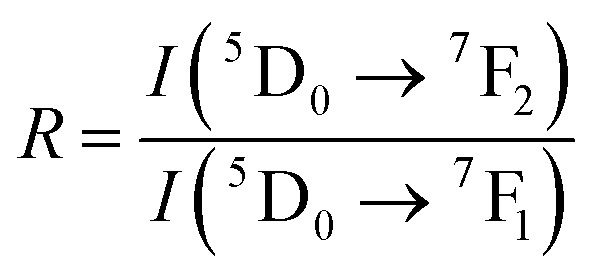


**Fig. 12:**
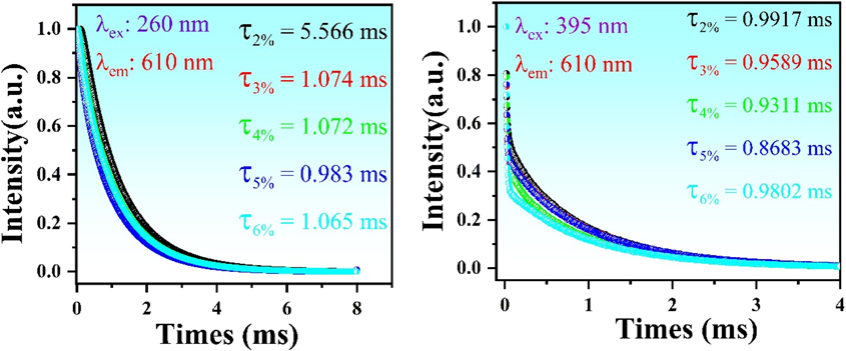
Time-resolved fluorescence spectra of CaY_2_O_4_ : *x*% Eu^3+^ samples calcined at 1200 °C.

The data in Table 3 show that as the Eu^3+^ ion concentration increases, the *R* ratio increases from 4.67 to 8.50 when the Eu concentration increases from 2 to 6%. This increase shows that the covalent character of the Eu–O bond increases in the Eu-doped CaY_2_O_4_ sample prepared by the solid-phase reaction method (Fig. 12).

### 3.6. The chromaticity coordinates and lifetime of the phosphor

To support the above conclusions regarding the influence of Eu^3+^ ion concentration on the optical properties of CaY_2_O_4_ material, we performed time-resolved fluorescence spectroscopy of CaY_2_O_4_ : Eu^3+^ samples with doping concentrations from 2 to 6% at different excitation wavelengths: 260 nm (corresponding to the charge transfer from O^2−^ to Eu^3+^ ions) and 395 nm (corresponding to the absorption of Eu^3+^ ions) for optimal emission. At the excitation wavelength of 260 nm, we observed that the fluorescence decay curve over time matched well with a double exponential function:3
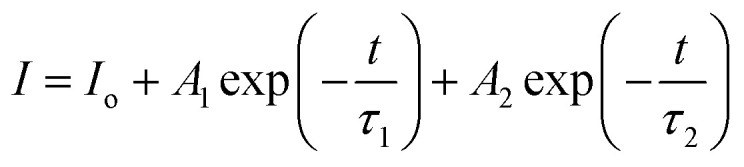


Meanwhile, at the excitation wavelength of 395 nm, the double exponential function could not be matched, requiring the use of a triple exponential function:4
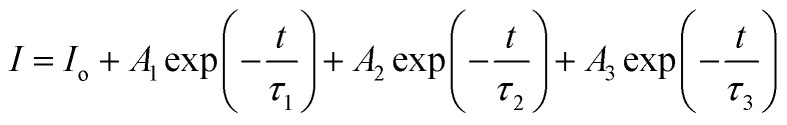
where *t* is the time, *I* (*t*) is the luminescence intensity at time *t*, *A*_1_, *A*_2,_ and *A*_3_ are constants, and *τ*_1_, *τ*_2_, and *τ*_3_ are exponential components of the decay time. The value of the average lifetime τ* can be calculated using the following formula (5):5
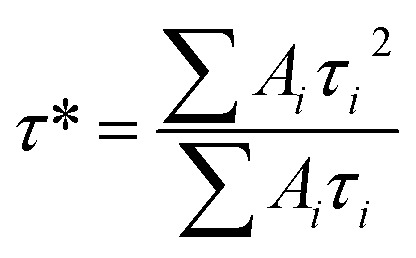


The analysis results in Table 2 show that when the Eu^3+^ ion concentration increases from 2–5%, the lifetime decreases from 0.9917 ms to 0.8683 ms.

**Table 2:** Theoretical lifetime, color coordinates, color temperature, and color purity of CaY_2_O_4_ samples doped with Eu at different concentrations

Eu^3+^ concentration	Average lifetime, τ* (ms)	Chromaticity diagram (*x*, *y*)		CCT (K)	Color purity (%)
2	0.9917	0.648	0.352	2415	95.36
3	0.9589	0.650	0.351	2526	96.62
4	0.9311	0.665	0.334	3313	99.72
5	0.8683	0.665	0.335	3307	99.73
6	0.9802	0.665	0.335	3307	99.73

The variation in lifetime can be interpreted in terms of concentration-dependent energy-transfer processes among Eu^3+^ ions in the CaY_2_O_4_ host lattice. As the Eu^3+^ concentration increases from 2 to 5%, more Eu^3+^ ions are incorporated into the host matrix, resulting in a higher density of luminescent centers and a shorter average distance between neighboring Eu^3+^ ions. The result favors energy migration among adjacent Eu^3+^ ions and increases the probability of non-radiative energy transfer from Eu^3+^ ions to lattice defects or quenching centers. Consequently, additional non-radiative relaxation channels are activated, leading to a faster depopulation of the excited ^5^D_0_ level and thus a gradual decrease in the fluorescence lifetime. This interpretation is consistent with the observed enhancement of emission intensity in CaY_2_O_4_ : Eu^3+^ samples as the Eu^3+^ concentration increases from 2 to 5%, where the increased number of active Eu^3+^ emission centers still dominates over the non-radiative quenching processes.

At 6% Eu^3+^ concentration, the emission intensity decreases, indicating the onset of concentration quenching. At this higher dopant concentration, the distance between neighboring Eu^3+^ ions becomes sufficiently small to promote stronger multipolar interactions and lead to a migration energy. The excitation energy can migrate through the Eu^3+^ sublattice and eventually be trapped by defects, distorted local environments, or other non-radiative quenching centers, where it is dissipated as lattice vibrations rather than emitted as photons. Interestingly, the lifetime shows a slight increase at this concentration. This behavior may be associated with the redistribution of excitation energy among different Eu^3+^ sites and with the increased contribution of slower-decaying emission centers to the measured decay profile as the rapidly quenched components become less dominant. In addition, the higher Eu^3+^ content may induce greater lattice distortion and modify the local crystal field around Eu^3+^ ions, which can further influence the balance between radiative and non-radiative relaxation pathways. Therefore, the simultaneous decrease in emission intensity and increase in lifetime at 6% Eu^3+^ should be considered an apparent lifetime effect arising from concentration quenching, site-dependent decay behavior, and energy trapping processes, rather than an improvement in overall luminescence efficiency.

To evaluate the color purity of the fabricated CaY_2_O_4_ : Eu^3+^ samples, we measured their color coordinates, color temperature, and color purity. The color purity of the materials was determined by the equation:6
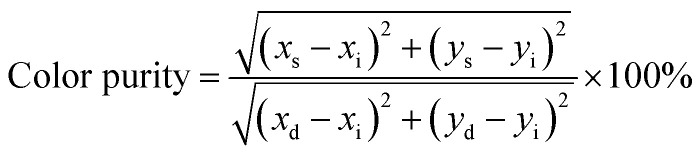


• (*x*_s_, *y*_s_): color coordinates of the fluorescent material sample to be measured.

• (*x*_i_, *y*_i_): standard white point coordinates (usually the CIE standard white point, *x*_i_ = 0.3101, *y*_i_ = 0.3162).

• (*x*_d_, *y*_d_): coordinates of the pure monochromatic color on the boundary of the CIE chromatogram with the same dominant wavelength as the sample.

Analysis results show that the Eu^3+^-doped CaY_2_O_4_ fluorescent material emits red light with high color purity, up to 99.7% for samples with Eu^3+^ doping concentrations of 4–6%. The results indicate that the fabricated material meets the requirements for manufacturing red-emitting LEDs with high monochromaticity, as well as for use as a red light supplement in white-emitting LEDs.

### 3.7. Judd–Ofelt parameters analysis

To better understand the changes in the crystal field structure around the Eu^3+^ ion as the Eu^3+^ ion concentration changes, we used J–O theory for the fluorescence spectrum of the Eu^3+^ ion to calculate the intensity parameters Ω_2_ and Ω_4_. As we know, the transitions from the ^5^D_0_ state to the ^7^F_*J*_ states (*J* = 2, 4, 6) are electric-dipole transitions of the Eu^3+^ ion, and these transitions are very sensitive to the environment surrounding the Eu^3+^ ion. Meanwhile, the dipole transition from the ^5^D_0_ state to the ^7^F_1_ state remains almost unchanged. The relationship between the intensity ratios of the transitions from the (^5^D_0_ – ^7^F_2,4,6_)/(^5^D_0_ – ^7^F_1_) states and the JO parameters (Ω_2_, Ω_4_, Ω_6_) is determined by the equation:7
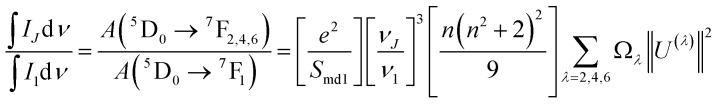


For the transitions ^5^D_0_ → ^7^F_2_, the matrix elements are *U*^(2)^ = 0.0033, *U*^(4)^ = *U*^(6)^ = 0. For the ^5^D_0_ → ^7^F_4_ transitions, *U*^(2)^ = 0, *U*^(4)^ = 0.0023, and *U*^(6)^ = 0. Lastly, for the ^5^D_0_ to ^7^F_6_ transitions, *U*^(2)^ = *U*^(4)^ = 0 and *U*^(6)^ = 0.003. The total area of the absorption bands for ^5^D_0_ to ^7^F_*J*_ (with *J* = 2, 4, 6) and ^5^D_0_ → ^7^F_1_ is also considered.^18^

The intensity parameters Ω_*λ*_ provide valuable insights into the local environment surrounding the Eu^3+^ ion. Analysis results show that as the Eu concentration increases from 2% to 5%, both values of Ω_2_ and Ω_4_ increase rapidly. This indicates that increasing the Eu concentration increases the asymmetry of the crystal field around the Eu^3+^ ion, thereby increasing the covalent bonding between the Eu^3+^ ion and the surrounding ligand, while also reducing the medium's stiffness as Ω_4_ increases.

The transition probability from the excited state *J* to a lower state *J*′ determines the fluorescence intensity of the *J* to *J*′ transition:8
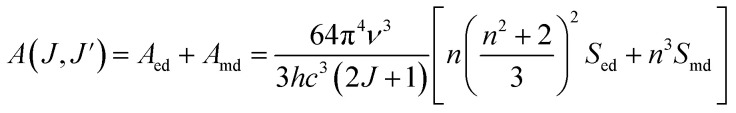


Total transition probability and lifetime of the excited state *J*:9
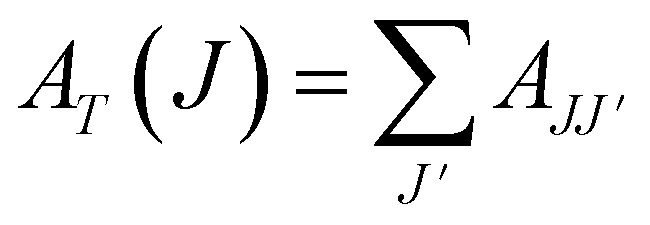
10
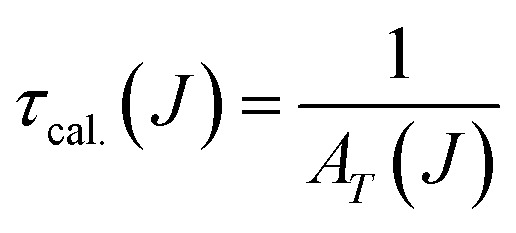


Branching ratio: used to predict the relative intensity of a fluorescence band from an excited state. The theoretical branching ratio is calculated using the formula:11
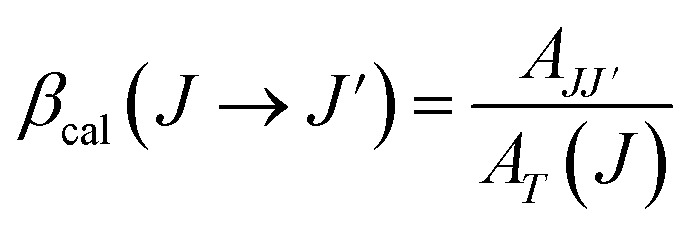


We used the branching ratio to predict the relative intensity of a fluorescence band from an excited state. The theoretical branching ratio is calculated using the formula:12
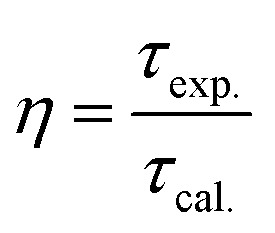


Quantum efficiency calculations of Eu-doped CaY_2_O_4_ materials show that the quantum efficiency ranges from 50.6% to 81.8% depending on the Eu doping concentration. The sample with the lowest quantum efficiency is the CaY_2_O_4_ sample doped with 2% Eu^3+^ ions; the quantum efficiency gradually increases to a maximum of 81.8% in the CaY_2_O_4_ : 5% Eu^3+^ sample and then decreases as the doping concentration increases above 5%. The results obtained based on the theoretical model are consistent with the fluorescence survey results. When the Eu concentration is low, energy transfer between Y^3+^ and Eu^3+^ ions is less efficient, leading to greater non-radiative recombination and lower luminescence. When the concentration of Eu^3+^ ions increases, the density of emission centers increases, leading to greater absorption and emission and, consequently, an increase in quantum efficiency. When the doping concentration reaches 5%, fluorescence quenching occurs, leading to a decrease in quantum efficiency. We compared these results with those of several other Eu^3+^-doped material systems to assess the suitability of the material we fabricated for monochromatic red-emitting LEDs and red-light SI for WLEDs. The comparison results are shown in Table 4.^27^

**Table 3:** Optical parameters Ω_2_, Ω_4_, intensity ratio *I*(^5^D_0_ → ^7^F_2_)/*I*(^5^D_0_ → ^7^F_1_), theoretical time, experimental lifetime, and intrinsic quantum efficiency of CaY_2_O_4_ samples doped with Eu at different concentrations

Samples	Ω_2_ (×10^−20^ cm^2^)	Ω_4_ (×10^−20^ cm^2^)	*R*	*τ*_cal_ (µs)	*τ*_exp_ (µs)	*η* (%)
CaY_2_O_4_ : 2% Eu^3+^	7.285	0.949	4.677	1919.5	991.7	51.66
CaY_2_O_4_ : 3% Eu^3+^	7.236	1.394	4.645	1893.4	958.9	50.66
CaY_2_O_4_ : 4% Eu^3+^	12.849	2.634	8.504	1147.3	931.1	81.2
CaY_2_O_4_ : 5% Eu^3+^	12.148	2.417	8.040	1059.5	868.3	81.8
CaY_2_O_4_ : 6% Eu^3+^	12.036	2.313	7.966	1222.7	980.2	80.2

**Table 4:** Comparison with some other red-emitting diodes doped with Eu^3+^ ions

Phosphor	*λ*_ex_ (nm)	*λ*_em_ (nm)	Colour purity (%)	CCT (K)	*η* (%)	Ref.
CaY_2_O_4_ : Eu^3+^	395, 465	612	99.73	3307	81.8	**This word**
CaY_2_O_4_ : Eu^3+^	393, 466	610	94.09	2229	—	18
ZnSnO_3_ : Eu^3+^	308, 532	614	—	—	—	23
CaF_2_ : Eu^3+^	395, 532	618	—	2368	—	24
SrY_2_O_4_ : Eu^3+^	254	611	—	2951	95.42	25
NiMoO_4_ : Eu^3+^	254	618	—	—	—	26
GdYGd : Eu^3+^	394	612	99.5	—	58.4	^27^
Y_3_TaO_7_ : Eu^3+^	394	611	94.7	—		28
SnNb_2_O_6_ : Eu^3+^	393	614	—	—	63.98	29
Ba_2_GdVO_6_ : Eu^3+^	395, 466	611	99.17	—	96.67	30
Ba_2_LaVO_6_ : Eu^3+^	395, 466	611	77.58	1926	22.02	30
Ba_2_SiO_4_ : Eu^3+^	394	614	—	—	68.2	31

## 4. Conclusions

We successfully fabricated Eu^3+^-doped CaY_2_O_4_ fluorescent materials with Eu^3+^ concentrations ranging from 2 to 6% using the solid-phase reaction method. The resulting materials exhibited an orthorhombic structure and crystallized best upon sintering at 1200 °C, with an average grain size of approximately 800 nm. As the Eu^3+^ ion concentration increased, the crystal size tended to decrease due to dopant inhibition at grain boundaries, increasing the likelihood of forming new crystal centers and reducing the average crystal size of the material at a constant sintering temperature. The material showed strong absorption of near-ultraviolet radiation (395 nm) and strong emission in the red region, with color purity up to 99.7% and a calculated quantum efficiency exceeding 80%. Concentration-induced fluorescence quenching was observed at a doping concentration of 5% Eu^3+^. XRD, SEM, EDX, FTIR, XPS, PL, PLE measurements, and J–O theory were applied to analyze the physical mechanisms of the material as sintering temperature and doping concentration varied. The analysis showed that the Eu^3+^-doped CaY_2_O_4_ material fabricated by the solid-phase reaction method is suitable for monochromatic red light-emitting LEDs with high color purity and for white LEDs (WLEDs).

## Author contributions

Pham Mai An: writing – original draft, methodology, investigation, formal analysis, data curation. Vu Thi Kim Lien: methodology, investigation. Can Ha Vi: investigation, formal analysis. Nguyen Thanh Binh: investigation, formal analysis. Ho Kim Dan: investigation, formal analysis, data curation. Chu Anh Tuan: methodology, investigation, conceptualization. Pham Thi Lien: investigation, formal analysis. Chu Viet Ha: formal analysis, methodology, investigation, data curation. Le Tien Ha: writing – review and editing, writing – original draft, methodology, investigation, data curation, conceptualization, funding acquisition.

## Conflicts of interest

The authors declare that they have no known competing financial interests or personal relationships that could have appeared to influence the work reported in this paper.

## Supplementary Material

RA-OLF-D6RA02573K-s001

## Data Availability

All data generated or analyzed during this study are included in this published article. No additional datasets were used or created. Additional data related to this paper may be requested from the corresponding author, halt@tnus.edu.vn. Supplementary information (SI) is available. See DOI: https://doi.org/10.1039/d6ra02573k.
