# Supplementary material for: Influence of sintering temperature and Eu3+ concentration on the structural, optical, and Judd–Ofelt parameters of CaY2O4 phosphors synthesized by solid-state reaction
Source: RSC Adv. 2026 Jul 2;16(34):32940–50. doi: 10.1039/d6ra02573k (PMC13326573; doi:10.1039/d6ra02573k)
Supplement: RA-016-D6RA02573K-s001 [file RA-016-D6RA02573K-s001.pdf]

Supplementary Information (SI) for RSC Advances.
This journal is © The Royal Society of Chemistry 2026

Supporting information

Influence of Sintering Temperature and Eu^{3+} Concentration on the Structural, Optical, and Judd–Ofelt Parameters of CaY_2O_4 Phosphors Synthesized by Solid-State Reaction

Pham Mai An[1], Vu Thi Kim Lien[2,3], Can Ha Vi[1], Nguyen Thanh Binh[1], Ho Kim Dan[4,5], Pham Thi Lien[6], Chu Anh Tuan[7], Chu Viet Ha[1], Le Tien Ha[7,*]

[1]Thai Nguyen University of Education, Thai Nguyen 250000, Vietnam

[2]Institute of Theoretical and Applied Research, Duy Tan University, Hanoi, 100000, Vietnam.

[3]Faculty of Natural Sciences, Duy Tan University, Da Nang, 550000, Vietnam.

[4]Optical Materials Research Group, Science and Technology Advanced Institute, Van Lang University, Ho Chi Minh City, Vietnam.

[5]Faculty of Applied Technology, Van Lang School of Technology, Van Lang University, Ho Chi Minh City, Vietnam.

[6]Institute of Materials Science, Vietnam Academy of Science and Technology, 18 Hoang Quoc Viet, Cau Giay, Hanoi, Vietnam

[7]Viet Nam University of Traditional Medicine, Hanoi, 100000, Vietnam.

[8]TNU - University of Sciences, Thai Nguyen 250000, Vietnam

Corresponding author's email: halt@tnus.edu.vn; letienha@tnu.edu.vn

3.S1. High-resolution transmission electron microscopy

To determine the crystal size and lattice plane spacing of the material more visually and accurately, we performed HR-TEM measurements on a 5%Eu^{3+}-doped CaY_2O_4 sample calcined at 1200 °C. The results in Figure S1.b show the SAED image of the material as concentric bright lines, revealing a polycrystalline structure with randomly arranged grains. The estimated crystal sizes from the SAED image corresponding to the lattice planes (023), (041), (130), and (150) are 2.99, 2.44, 1.95, and 1.73 Å, respectively. Figure S1.c is an HR-TEM image of this sample, and we estimate the interplane spacing in this image to be approximately 2.95 Å, which is the interplane spacing (023) of the CaY_2O_4 material. The result is consistent with the crystal size analysis through the Scherrer formula.

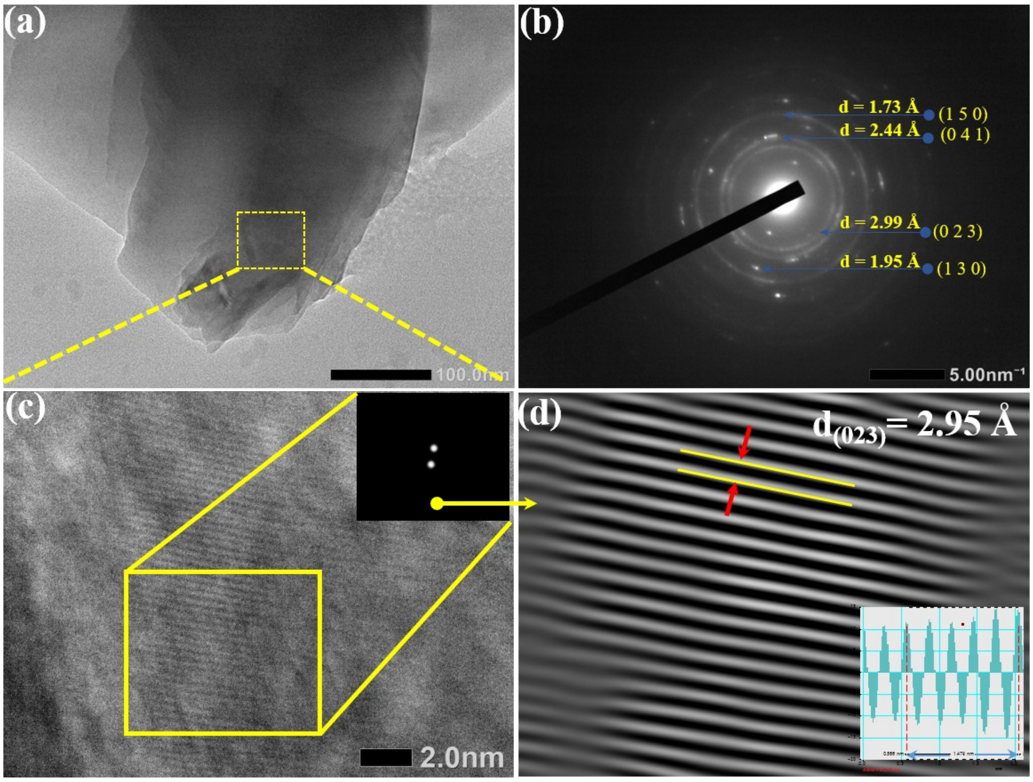

Fig S1. HR-TEM and SAED images of the CaY_2O_4:5%Eu sample calcined at 1200°C.

3.S2. Thermal stability of materials

To evaluate the applicability of the fabricated fluorescent material for LED coating, its thermal stability is crucial. To investigate this issue, we measured the fluorescence spectrum of CaY_2O_4:5%Eu^{3+} material, which exhibits the best luminescence from room temperature to 210 °C under 395 nm excitation. Analysis showed that the positions of the material's emission peaks remained almost

unchanged, while the fluorescence intensity decreased gradually with increasing temperature. This result can be explained by the fact that, as temperature increases, the thermal vibrations of the molecules become stronger, leading to an increase in the number of phonons in the material's crystal lattice, thereby enhancing multi-phonon expansion and facilitating energy transfer between the Eu^{3+} luminescent centers and non-radiative quenching sites. Therefore, a portion of the excitation energy is dissipated through lattice vibrations rather than recombination, leading to emission and thus reducing the fluorescence intensity. To evaluate the temperature-induced fluorescence attenuation and determine the activation energy for fluorescence quenching, we applied Arrhenius theory using the following equation:

$$I(T) = \frac{I_0}{1 + C.\exp\left(-\frac{E_a}{k_B T}\right)} \tag{S1}$$

where I(T) is the fluorescence intensity at temperature T; I_0 is the fluorescence intensity at initial room temperature; E_a is the thermal quenching activation energy; k_B = 8.6173 eV/K is the Boltzmann constant.

Rearranging the above equation, we get:

$$\frac{I_o}{I(T)} = 1 + C.\exp\left(-\frac{E_a}{k_B T}\right) \Rightarrow \ln\left(\frac{I_o}{I(T)} - 1\right) = \ln C - \frac{E_a}{k_B T} \tag{S2}$$

Based on the results of the temperature dependence of fluorescence intensity, we plotted ln(I0/I-1) and 1/kBT and performed linear interpolation. The obtained E_a value for this material is 0.106 eV. This result shows that the fluorescence decay is mainly due to heat-activated non-radiative recombination, which becomes more pronounced with increasing temperature.

The analysis results show that when the material temperature reaches 150 °C (approximately 410 K), the sample's fluorescence intensity still retains about 66% of its value at room temperature. These results indicate that the $CaY_2O_4:Eu^{3+}$ material meets the requirements for use as a fluorescent material for red-light emitting LEDs, as well as a complementary material for white LEDs (WLEDs) to produce warm white light.

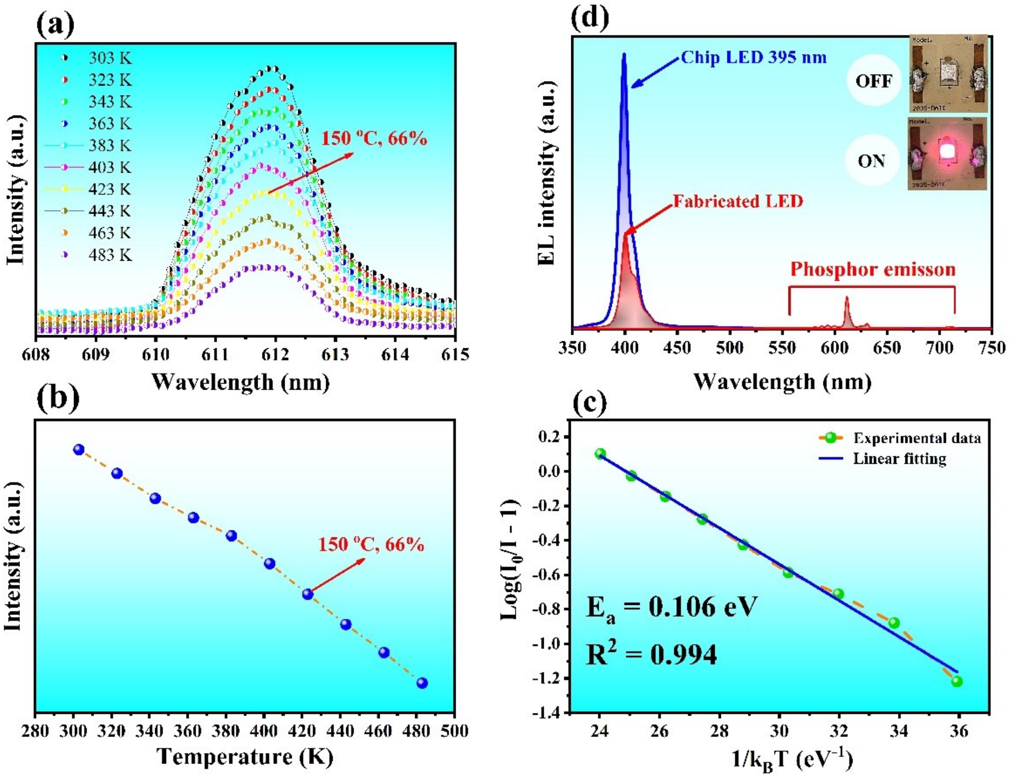

Fig S2. (a) Temperature-dependent PL spectra of the CaY_2O_4:5%Eu^{3+} phosphor. (b) Normalized emission intensity of CaY_2O_4:5%Eu^{3+} fluorescent powder at different temperatures. (c) The graph shows the relationship between Log(Io/I-1) and $1/k_BT$, and (d) EL spectra of the fabricated LED with and without the optimized phosphors layer.
